# K2 crew performance: a preliminary investigation of kinetic parameters in preferred and inverted positions among sub-elite kayakers

**DOI:** 10.3389/fphys.2024.1498111

**Published:** 2024-11-19

**Authors:** Cristian Romagnoli, Saeid Edriss, Lucio Caprioli, Luca Ghelardini, Ida Cariati, Anas Alashram, Nunzio Lanotte, Paolo Boatto, Elvira Padua, Vincenzo Bonaiuto, Giuseppe Annino

**Affiliations:** ^1^ Department of Human Science and Promotion of Quality of Life, San Raffaele Open University, Rome, Italy; ^2^ Sports Engineering Laboratory, Department of Industrial Engineering, University of Rome Tor Vergata, Rome, Italy; ^3^ Coach of Italian Canoe/Kayak Federation (FICK), Rome, Italy; ^4^ Department of Medicine Systems, University of Rome Tor Vergata, Rome, Italy; ^5^ Department of Physiotherapy, Faculty of Allied Medical Science, Middle East University, Amman, Jordan; ^6^ APLAB, Roma, Italy; ^7^ Human Performance Laboratory, Centre of Space Bio-Medicine, Department of Medicine Systems, University of Rome Tor Vergata, Rome, Italy

**Keywords:** water sports, E-kayak system, performance analysis, flatwater kayak, crew, paddling

## Abstract

K2 performance depends on different kinematic and kinetic variables. Due to the lack of related studies in this area, we have tried to explain these features to better understand the best positioning of paddlers and how their synchronization affects performance. This study uses the DAQ system comprising two instrumented paddles—an IMU and a GPS (“E-kayak” system)—to investigate paddle synchronization and the specific positioning of paddlers’ in preferred and inverted configurations. In this study, 10 sub-elite paddlers participated, divided into five crews. The test included two trials of 500 m performed in preferred and inverted seating positions. The synchronization analysis highlighted that the rear paddler contributed efficiently to the propulsion of the boat while performing 30–40 ms earlier than the front paddler during the entry and exit phases. Despite the time results for 500 m, there is no evidence indicating a dominant indication of the preferred or inverted position among the athletes. The results show a significant correlation (p < 0.05) between the force of the front paddler (r = −0.88), the stroke frequency of the crew (r = −0.66), and the total force applied by the crew with the time for 500 m and between stroke frequency and the force of the front paddler (r = 0.64). Based on these indications, for only those crews who completed the 500 m test in the shortest time, the equation determining the time over 500 m was calculated using multiple regression analysis, considering the stroke frequency and the force of the front and rear paddler. The data showed a good estimation with CV% = 0.22, ICC = 0.99, and ES = −0.005. In conclusion, these findings can serve as a beneficial tool for assessing or monitoring K2 crew performance in sub-elite paddlers.

## Introduction

Flatwater sprint kayaking is an Olympic discipline with individual (K1) and team competitions (K2 and K4) held over distances of 200, 500, and 1,000 m. In K2 kayaking, two athletes sit in tandem and, through cyclic and synchronized paddle movements, generate the propulsive force needed to overcome water resistance and accelerate the kayak (Romagnoli et al., 2022a; Romagnoli et al., 2022b). The synchronization between the athletes (referring to the temporal coordination of the different phases of their paddling cycle) is influenced by the kayak’s design and the athletes’ paddling timing (King and de Rond, 2011; Tay and Kong, 2018). This aspect plays a crucial role in avoiding paddle contact (Tay and Kong, 2018). Correct synchronization has been identified as a critical determinant of performance in kayak sprinting (Robinson et al., 2011; Bonaiuto et al., 2022; Romagnoli et al., 2024) and rowing (Wing and Woodburn, 1995; King and de Rond, 2011; Cuijpers et al., 2015). Although Kong et al. (2020b) suggested that a well-synchronized crew maintaining the same paddling rhythm can enhance performance, several studies indicate that slight asynchrony may be even more beneficial (Martin and Bernfield, 1980; Tellez et al., 2015). Tellez et al. (2015) showed that some of the best sprint kayak crews exhibit a slight asynchrony in paddling (with the rear paddler starting the stroke earlier and finishing it later than the front paddler). According to Martin and Bernfield (1980), this asynchrony could reduce power loss caused by fluctuations in kayak speed in the forward direction. However, studies supporting this theory have mainly been conducted on rowing ergometers (Brouwer et al., 2013) and through 2D video analysis (Tay and Kong, 2018). The latter research revealed that crews in K2 tend to be more synchronized during the catch than the release phase of paddling. Furthermore, paddling synchronization patterns vary significantly, and sprint kayakers have no universal synchronization profile. It is complex to study the coordination between crew members and their interactions with the boat and surroundings. Another crucial aspect of performance in K2 is the athletes’ positioning. Although there is a position that is generally considered optimal, current literature offers divergent opinions on what it should be. For example, Ong et al. (2005) and Tellez et al. (2015) argued that taller and heavier athletes should sit at the rear, as they are more effective in generating paddling force. In contrast, through a computational fluid kinetics analysis, Campbell Ritchie and Selamat (2010) found that the load experienced by the front athlete is greater than the rear and suggested locating the stronger athlete in front. According to Tay and Kong (2020), the common practice is to place the powerful paddler in the back (rear), showing that four out of eight assessed crews achieved better times by reversing the seating order from that preferred. Kong et al. (2020a) also found no differences in strength, power, or time variables between front and rear paddlers, even when athletes switched positions. The literature has not reported any useful indication for crew composition. Based on these considerations, the present study aims to investigate the paddling timing in K2 crews, analyzing the advances or delays between front and rear kayakers during the paddle entry and exit phases (Romagnoli et al., 2022a) and the best location of the athletes in the kayak, measuring kinematic and kinetic parameters through the use of the “E-kayak” system (Bonaiuto et al., 2020; Bonaiuto et al., 2022).

## Materials and methods

### Subject

A total of 10 male sub-elite kayakers aged 15.2 ± 0.42, height 171.6 ± 3.92 (cm), mass 61.45 ± 4.79 (kg), and a kayaking experience of 4.80 ± 1.23 (years) participated in the study (Table 1). They were national-level athletes from the Canottieri Eur club who trained nine times a week during the test periods. The study was approved by the University of Rome Tor Vergata Institutional Review Board. Testing procedures were fully explained to participants’ families before obtaining individual written informed consent. All procedures followed the Declaration of Helsinki.

**TABLE 1:** Anthropometric characteristics of each paddler.

Paddler	Age (yrs)	Height (cm)	Lower limb length (cm)	Arm length (cm)	Trunk length (cm)	Kayak experience (yrs)
A1	15	175	105	75	36	4
A2	15	178	107	80	37	7
B1	15	170	103	73	37	3
B2	16	169	102	73	40	5
C1	15	174	105	76	41	6
C2	15	168	105	77	40	6
D1	16	179	108	80	39	4
D2	15	168	97	72	35	4
E1	15	168	100	75	38	4
E2	15	167	103	73	35	5
Mean ± SD	15.2 ± 0.42	171.6 ± 4.50	103.5 ± 3.27	75.4 ± 2.87	37.8 ± 2.15	4.8 ± 1.23

### Test procedure

The test trials were all conducted early in the morning (between 7 a.m. and 9 a.m.) when the lake conditions were flat, not windy, and suitable for this study (weather temperature at approximately 22 °C and water temperature at approximately 18 °C). The athletes were asked to race with their optimal performance in two 500 m trials following the coach’s directions through the race records and then in inverted positions.

Before each test, the paddlers performed a 15-min warm-up on land (focusing on shoulder and pelvic joint mobility exercises) and a more intensive warm-up in a K2 craft for up to 20 min. On the first day of the test, three crews performed the simulated race in their preferred positions and two in inverted positions, while on the second test day (after one recovery day), all crews switched their positions. Each trial was monitored through the E-kayak system, which consisted of an IMU (TDK 20948-100 Hz) and a GPS (ublox NEO-M9N-25 Hz) placed behind the rear paddler seat; two paddles instrumented with a strain gauge (100 Hz) were used (Bonaiuto et al., 2022).

### Variable extraction

**FIGURE 1:**
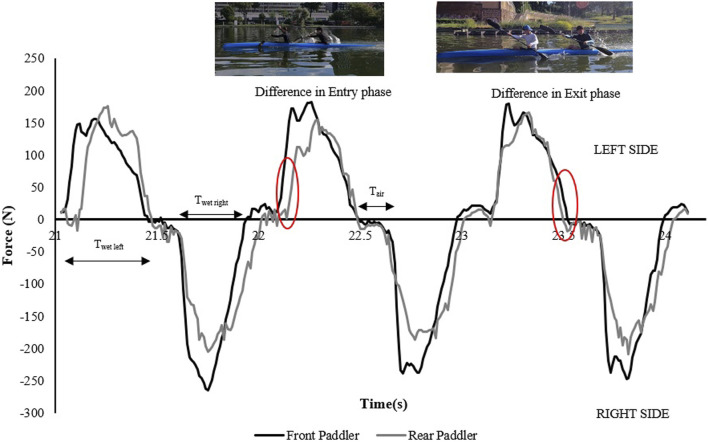
Force–time curves of a K2 crew showing the air and wet time (T_air_–T_wet_) for each side and highlighting the time difference during the paddle entry and exit phases between front and rear kayakers.

The following kinematic and kinetic parameters were measured for each crew in the preferred and inverted mode: the air or aerial phase (from the paddle blade exit to the entry on the other side), the wet phase (from water entry to exit for each paddle blade) (McDonnell et al., 2012; Gomes et al., 2015; Bonaiuto et al., 2020), the average force for front and rear paddlers, the total force expressed from each crew, the stroke frequency, and the total time over 500 m. The definition of the entry and exit phases of the paddle corresponded to the start and end of force application in water (Figure 1). Figure 1 shows part of the force–time plot of a crew with the respective advances or delays between the front and rear kayakers during the entry and exit phases. All parameters considered were extrapolated and analyzed from the data provided by E-kayak (Romagnoli et al., 2024).

### Statistical analysis

The results are presented as the mean (M) and standard deviation (±SD) unless otherwise specified. The Kolmogorov–Smirnov test was used to validate the assumption of normality. To verify the correlation between time over 500 m and each crew’s kinematic and kinetic data, the Pearson product–moment correlation coefficient (r) and 95% confidence interval (95% CI) for r were used. Statistical significance was accepted at *p* < 0.05. A multiple regression model was used to quantify the relationship $Y=\left(K_{0}+K_{1}X_{1+}K_{2}X_{2+}K_{3}X_{3}+K_{n}X_{n}\right)$ between the dependent variable (*Y*=Time on 500 m) and a set of explanatory variables (stroke frequency (*X*_1_), force of front paddler (*X*_2_), and force of rear paddler (*X*_3_)), along with respective variance of the faster crew at 500 m. A weight dummy variable was assumed (AutoWeight 1/SD^2^) for an automatic weighted regression procedure to correct for heteroscedasticity (Neter et al., 1996). In addition, the coefficient of variation percentage (CV%), the effect size (ES), where a small effect was 0.1, a moderate effect was 0.3, and a large effect was 0.5 (Cooper and Hedges, 1993), and the relative 95% CI were calculated for the time measured at 500 m and the time estimated through the multiple regression equation. Furthermore, the interclass correlation (ICC) was used to assess reliability between the time measured and time estimated from the multiple regression equation. The statistics and data visualization were performed using MedCalc^®^ (version 23.0.1.).

## Results

If the mean value is positive, the rear always anticipates the front paddler during the entry and exit phases. If the mean value is negative, the rear paddler postpones the entry and exit relative to the front paddler. These differences in both phases highlight the different paddling techniques adopted by each crew (Table 2).

**TABLE 2:** Average differences and relative standard deviation of the blade entry and exit phases in the water between front and rear paddlers.

Crew	Difference rear-front (entry phase) (s)	Difference rear-front (exit phase) (s)	Total time over 500 m (s)
**A1**–**A2 (preferred)**	0.033 ± 0.027	0.040 ± 0.023	**105.568#**
A2–A1 (inverted)	−0.004 ± 0.031	−0.001 ± 0.041	118.120
**B1**–**B2 (preferred)**	0.017 ± 0.021	−0.023 ± 0.035	**109.328#**
B2–B1 (inverted)	0.026 ± 0.044	0.091 ± 0.074	118.372
**C1**–**C2 (preferred)**	0.002 ± 0.021	0.016 ± 0.030	**122.862#**
C2–C1 (inverted)	0.006 ± 0.024	0.007 ± 0.028	127.297
D1–D2 (preferred)	0.064 ± 0.019	0.027 ± 0.021	109.204
**D2**–**D1 (inverted)**	−0.009 ± 0.025	0.035 ± 0.021	**108.955#**
E1–E2 (preferred)	0.027 ± 0.030	0.040 ± 0.024	114.632
**E2**–**E1 (inverted)**	−0.017 ± 0.026	−0.027 ± 0.026	**114.085#**

# represent the fast trial performed in preferred or inverted crew. The bold values together with # highlight the best performance for each crew.

Table 3 shows the correlations and relative 95% CI between time over 500 m, the average total force developed by front and rear paddlers, front and rear paddlers’ strength, and stroke frequency. The total analysis of the crews in both the preferred and inverted positions shows a significant correlation between the time over 500 m and the average total force developed by the crew, the strength of the front kayaker, and the stroke frequency of K2. These results suggest that the front paddler plays a fundamental role in leading the crew by imposing the stroke frequency. No statistically significant correlations were found for all other parameters considered in the study (*p* > 0.05).

**TABLE 3:** Time over 500 m correlated with the force expressed by front and rear kayakers, total force of crew (front + rear), stroke frequency, and 95% CI.

Variable	Front force (N)	Rear force (N)	Total force (front + rear) (N)	Stroke frequency (s/min)
Time on 500 m (s)	r = −0.88 95% CI = −0.97 to −0.56; p = 0.0008**	r = −0.47 95% CI = −0.85 to 0.22; p = 0.16	r = −0.78 95% CI = −0.94 to −0.29; p = 0.008**	r = −0.66 95% CI = −0.91 to −0.057; p = 0.037*

Significance is reported as *p < 0.05 and **p < 0.01.

Table 4 shows the correlations between stroke frequency, front and rear paddlers’ strength, and time over 500 m. The only statistically significant correlations were between front kayaker strength and time over 500 m.

**TABLE 4:** Stroke frequency correlated with force expressed by front and rear paddlers and time over 500 m.

Variable	Front force (N)	Rear force (N)
Stroke frequency (s/min)	r = 0.64 95% CI = 0.017 to 0.90; p = 0.046*	r = −0.02 95% CI = −0.64 to 0.62; p = 0.95

Significant is reported as *p < 0.05.

The results from multiple regression are shown in Table 5. It indicates that the analysis of the variance table separates the total variation in the dependent variable into two parts: one attributed to the regression model (labeled “Regression”) and another that cannot be explained by the model (labeled “Residual”). If the *p*-value for the F-test is small (less than 0.05), as in the results obtained (F-ratio = 224.84 and *p*-value = 0.049), the hypothesis of no (linear) relationship can be rejected, and the multiple correlation coefficient is considered statistically significant. The equation takes account of the front and rear force of paddlers and the K2 stroke frequency; these parameters seem to determine the time over 500 m for the fast crews.

**TABLE 5:** Summary of multiple regression equations and analysis of variance. The weighted least squares multiple regression section reports the coefficient of determination R^2^, R^2^ adjusted, multiple correlation coefficient (MCC), and residual standard deviation (RSD). The regression equation section shows independent variables and relative coefficient (K), standard error (Std. Er), 95% CI of K, t-value (t), P-value (P), and partial correlation (r_partial_). Analysis of variance section shows F-ratio and P-value (P).

Analysis of variance	
Weighted least squares multiple regression	
Crew (best time)	5
Coefficient of determination R^2^	0.998
R^2^-adjusted	0.994
MCC	0.999
RSD	2.060

Regression equation						
Independent variable	Coefficient	Std. Er	95% CI	t	P	r_partial_
(Constant)	197.7676	6.95	109.43 to 286.09	28.44	0.022	—
Stroke_frequency (SF)	−0.2168	0.092	−1.39 to 0.95	−2.34	0.256	−0.919
ForceFront_paddler (F_f_)	−0.8158	0.072	−1.73 to 0.10	−11.21	0.056	−0.996
Force Rear_paddler (F_r_)	0.3007	0.030	−0.087 to 0.68	9.83	0.064	0.994

**FIGURE 2:**
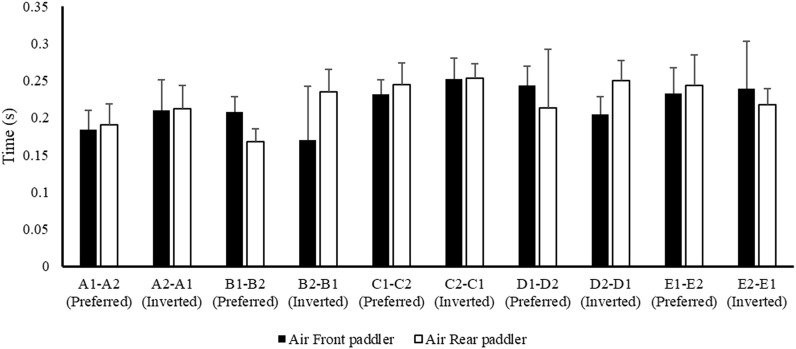
Mean value ± SD of the air phase of the front (black) and rear (white) paddlers of each crew during the 500 m test.

**FIGURE 3:**
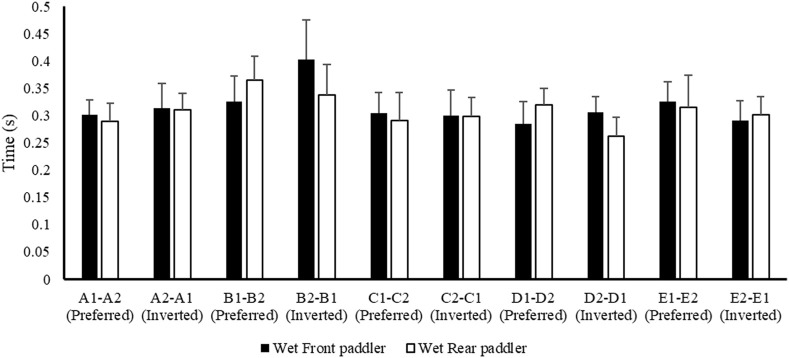
Mean value ± SD of the wet or water phase of front (black) and rear (white) paddlers during the 500 m test.

**FIGURE 4:**
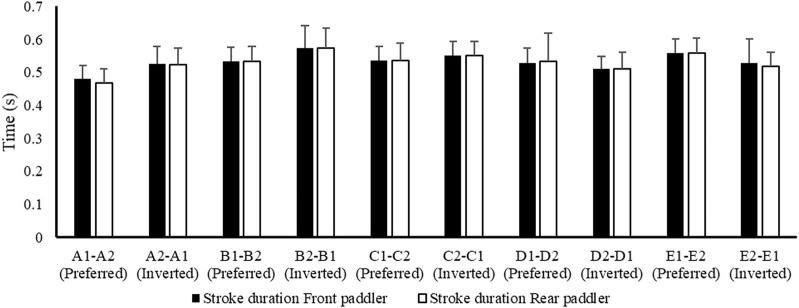
Average values ± SD of stroke duration between front and rear paddlers shown in preferred and inverted positions during the 500 m test.

Figure 2 shows the mean data of air phase duration between the front and rear paddlers for each crew in the preferred reversed position, while Figure 3 shows the average data of the water phase for each component of the crew. Figure 4 reports the mean duration of paddle strokes (T_air_ + T_wet_), considering the air and water phases of each athlete (for each stroke) in different seats during their performance.

**FIGURE 5:**
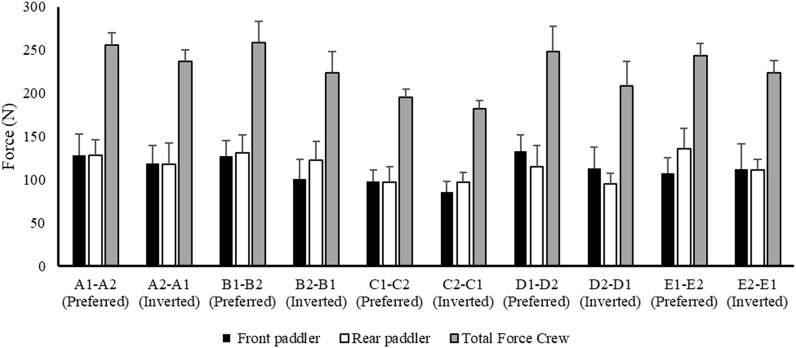
Mean value ± SD of force related to front (black) and rear paddler (white) and total force of the crew (gray).

Figure 5 reports the data related to force expressed during the water phase of the front and rear paddlers in preferred and reversed seats. The total force (front + rear) provides a better understanding of the total mean force applied during the 500 m.

**FIGURE 6:**
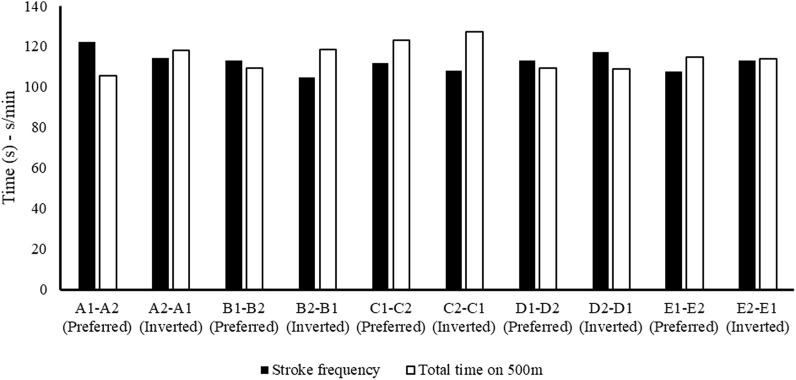
Black-and-white histogram representing, respectively, the stroke frequency (s/min) and total time (s) performed over 500 m in the preferred and inverted seating positions.

Figure 6 reports the stroke frequency (black) and time performed on 500 m (white) in preferred and inverted seats for each crew. The figure shows that the crew with a high stroke frequency obtains the best performance.

These results (Figures 2–6) show the different strategies for each crew in preferred and inverted positions to lead the 500 m at maximum velocity, adapting the kinematic and kinetic parameters and the technical gesture.

**FIGURE 7:**
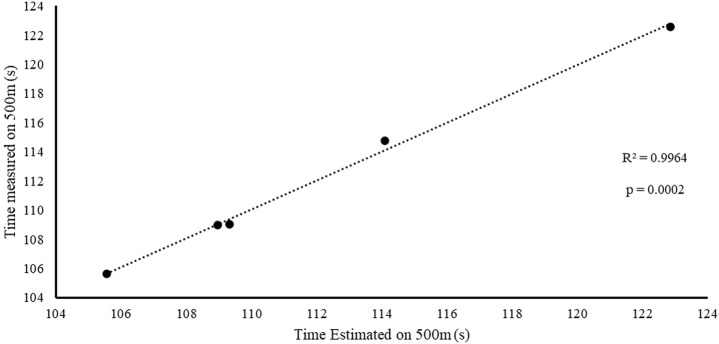
Correlation between the measured and estimated times (from the multiple regression equation) over 500 m for the fastest crews only (A1–A2; B1–B2; C1–C2; D2–D1; and E2–E1).

Figure 7 reports the correlation between time measured over 500 m and the time estimated by the multiple regression model for the five fastest crews (A1–A2; B1–B2; C1–C2; D2–D1; and E2–E1). The results show a high correlation between the two (r = 0.99), with CV% = 0.22, ES = −0.005, and ICC = 0.99. This further confirms Table 5, which shows that the parameters related to front and rear paddlers’ strength and relative stroke frequency are critical for crews in K2.

## Discussion

Chronometric analysis of the 500 m in K2 performed in the preferred and inverted orders shows that three of the crews performed the fastest run in the preferred position and two in the inverted position (Table 2). The data agree with the findings of Tay and Kong (2020) that approximately 50% of analyzed crews achieved better times by reversing the seating order from the preferred order. Moreover, they pointed out that the seating order in K2 minimally affects the synchronization of strokes and is not always decisive for maximum performance. Our tests show that the key to better performance is the paddling timing during the entry and exit phases of the water of the two athletes, also considering the levels of force and stroke frequency they express.

From the analysis in Table 2, the crew with the best performance over 500 m is in the preferred position A1–A2. During the entry and exit phases, the rear paddler always anticipated the entry and exit of the blade with an average timing difference between 33 and 40 ms. This temporal asynchrony confirms the data reported by Gomes (2015) and Kong et al. (2020a), who reported an anticipation of 34 ms during the entry phase. This anticipation explains that during the phase of blade entry into the water, the rear paddler must react quickly to generate high force values on the blade to compensate for the higher speed of the fast-moving water near the boat, and an early exit minimizes the drag (i.e., the deceleration of the K2 that starts before the end of the exit phase). The analysis showed an average timing offset of 38.30 ms and 41.70 ms in the early and exit phases, respectively. In contrast, for the other crews investigated in both the preferred and inverted positions, the kinematic data fall outside the ranges identified, with a minimum of −17 ms and a maximum of 64 ms in the entry phase (Gomes, 2015; Kong et al., 2020a). In the exit phase, the time ranged from a minimum of −27 ms to a maximum of 91 ms. This variation may have influenced the crew’s propulsive action. For example, in the B1–B2 crew (preferred position), an average anticipation of 17 ms in the entry phase and a delay of −23 ms in the exit phase by the rear paddler were observed. This difference in timing during water force application, where the rear paddler starts the stroke earlier and finishes later, could help reduce the power loss of the K2 during the advancement (Martin and Bernfield, 1980; Tellez et al., 2015). In addition to the timing of force application in the water, other kinematic and kinetic factors also appear to influence the performance of the K2 500 m. Figures 2–4 show the plots of the average durations of the air and wet phases and the stroke duration (T_air_ + T_wet_) of the front and rear paddlers in both the preferred and inverted positions. The statistical analysis showed a non-statistically significant correlation (*p* > 0.05) between the stroke duration (T_air_ + T_wet_) and 500 m performance, with a correlation of 0.62 for the front paddler and 0.60 for the rear paddler. Nevertheless, this result could be statistically significant in elite athletes, whose superior paddling techniques allow for a more efficient application of propulsive force. In elite paddlers, the air phase is not excessively long, minimizing deceleration due to drag. Another important aspect is the average and total forces exerted by the front and rear paddlers during 500 m in both the preferred and inverted positions. Figure 5 shows the average force values and their respective sums between the front and rear paddlers. The analysis shows that the time over 500 m is correlated with the sum of the force exerted by the crew, so the stronger crew (able to generate better propulsive power) is that which covers 500 m in less time (Table 3). This aspect is relevant because expressing a high level of power requires high levels of force and paddle velocity during the propulsive phase (Kristiansen et al., 2022; Romagnoli et al., 2022b). Another important aspect is the correlation found between the front paddler’s strength and time over 500 m (Table 3). This correlation, r = −0.88, seems to confirm the findings of Campbell Ritchie and Selamat (2010), where the maximum load experienced on the blade is found for the front athlete. This aspect could be explained because, in sub-elite crews, the more technical athlete is positioned in front and is consequently able to express a greater force than those with less technique. Furthermore, it is relevant that in all tests, the paddling frequency affected the total time. In particular, the analysis shows that the best test (between preferred and inverted) (Figure 6) is always that with a higher paddling frequency, as demonstrated by athletes in K1 (Caldognetto and Annino, 2010). Finally, a significant correlation is observed between front paddler strength and paddling frequency (Table 4). This could be further confirmation that for sub-elite athletes in K2, the front paddler must be the most technical. Consequently, they are able to develop high force gradients and thus dictate the optimal paddling frequency for the crew throughout the performance. Considering the impact that these features can have on K2 crews, a specific regression equation is investigated. From the results in Table 5, the analysis confirms the goodness-of-fit of the model, with R^2^ = 0.99 (indicating the proportion of the variation in the dependent variable explained by the regression model) and MCC = 0.99. Despite t and P values in the regression equation section being not significant, we can consider the independent data (stroke frequency, force front paddler, and force rear paddler) to be statistically significant to estimate the dependent data (time over 500 m) because the relative F-ratio and P-value of analysis of variance is less than 0.05 (Altman, 1990; Armitage et al., 2013). From the comparison between the time measured and estimated over 500 m (Figure 7), CV% is 0.22 (95% CI from 0.00 to 0.38), ICC is 0.99 (95% CI from 0.98 to 0.99), and ES is −0.005 (95% CI from −0.11 to 0.05). For this reason, the following multiple regression equation applies:

$$Time\;on\;500m=197.77+\left[\left(-0.217\;SF\right)+\left(-0.816\;F_{f}\right)+\left(0.301\;F_{r}\right)\right],$$

where SF is the stroke frequency, F_f_ is the force of the front paddler, and F_r_ is the force of the rear paddler.

This formula could be used by trainers to investigate how these parameters can influence the time over 500 m for sub-elite paddlers, and it could also serve as a valid tool for selecting a better K2 crew.

## Limitations

The following study has some limitations, including the sample size of the crews, the technical level of the kayakers (sub-elite), the absence of an instrumented footrest to measure lower limb force, and the lack of a reference model (elite crew). With elite paddlers, parameters such as the water and air phases could play a fundamental role in K2 crew performance. For these reasons, future research is needed to understand whether other variables should be taken into consideration as dependent variables in multiple regression equations to explain K2 crew performance.

## Conclusion

This study was conducted on sub-elite K2 athletes, and no significant differences were observed in 500 m race outcomes between the five crews in the preferred/inverted positions, with three performing best in the preferred session and two in the inverted position. However, a minimal difference of a few tenths confirms that there is no predominant position, as also observed by Kong et al. (2020a). The key findings emerging from the analysis suggest that the front paddler plays a crucial role in the crew’s performance as they must be able to produce a high level of force in the water while simultaneously maintaining an optimal paddling frequency for performance purposes. Furthermore, the rear paddler must be able to anticipate the front paddler during the entry and exit phase by approximately 30–40 ms to efficiently contribute to the propulsion of the boat, as previously observed by Gomes (2015), Kong et al. (2020a). Finally, the individualized multiple regression equations developed in this study can serve as valuable tools for assessing and monitoring different kinematic and kinetic parameters of sub-elite K2 crew performance. E-kayak software makes it possible to investigate kinetic parameters among the paddler’s crews to individualize some K2 performance limiting factors. Consequently, future studies could investigate elite crews to determine a performance model for K2 racing. Finally, the future integration of E-kayak with emerging artificial intelligence technologies could provide valuable tools for further investigating these concepts, including paddlers’ pose estimation and object detection (Edriss et al., 2024b; Edriss et al., 2024a; Najlaoui et al., 2024).

## Data Availability

The raw data supporting the conclusions of this article will be made available by the authors, without undue reservation.
